# Interfacial-energy-directed synthesis of mesoporous COF architectures

**DOI:** 10.1093/nsr/nwag454

**Published:** 2026-07-27

**Authors:** Jing Tang, Yusuke Yamauchi

**Affiliations:** State Key Laboratory of Petroleum Molecular & Process Engineering, Shanghai Key Laboratory of Green Chemistry and Chemical Processes, School of Chemistry and Molecular Engineering, East China Normal University, China; Australian Institute for Bioengineering and Nanotechnology (AIBN) and School of Chemical Engineering, The University of Queensland, Australia; Department of Materials Process Engineering, Graduate School of Engineering, Nagoya University, Japan

Covalent organic frameworks (COFs) represent a landmark in reticular chemistry, offering crystalline porous polymers constructed from organic building blocks through reversible covalent bonds [1]. Their tunable pore sizes, diverse framework compositions, tailorable microenvironments and high structural stability have positioned COFs as versatile platforms for energy storage and conversion, catalysis, and separation [2]. However, most COFs remain confined to the micropore regime (<2 nm), which severely limits the accessibility of active sites and impedes mass transport. Constructing mesoporous COFs (MesoCOFs) with large, open channels has therefore emerged as a major objective in COF research.

The challenge, however, lies in the intrinsic nature of COF crystallization. COF precursors exhibit a strong tendency toward rapid self-growth, which competes with the cooperative assembly of surfactant micelles and COF precursors. Conventional strategies to overcome this barrier, such as relying on strong intermolecular interactions, e.g. ionic interactions, have imposed substantial limitations on MesoCOFs development, particularly in compositional diversity and precise structural modulation. Recently, kinetics-mediated micelle assembly has emerged as a promising avenue, in which the reaction kinetics is shifted into a non-classical growth regime to enable cooperative self-assembly between COF precursors and micelles [3]. Nevertheless, systematically controlling symmetry, mesophase and pore size while translating this structural diversity into improved performance remains a formidable challenge.

**Figure 1.**
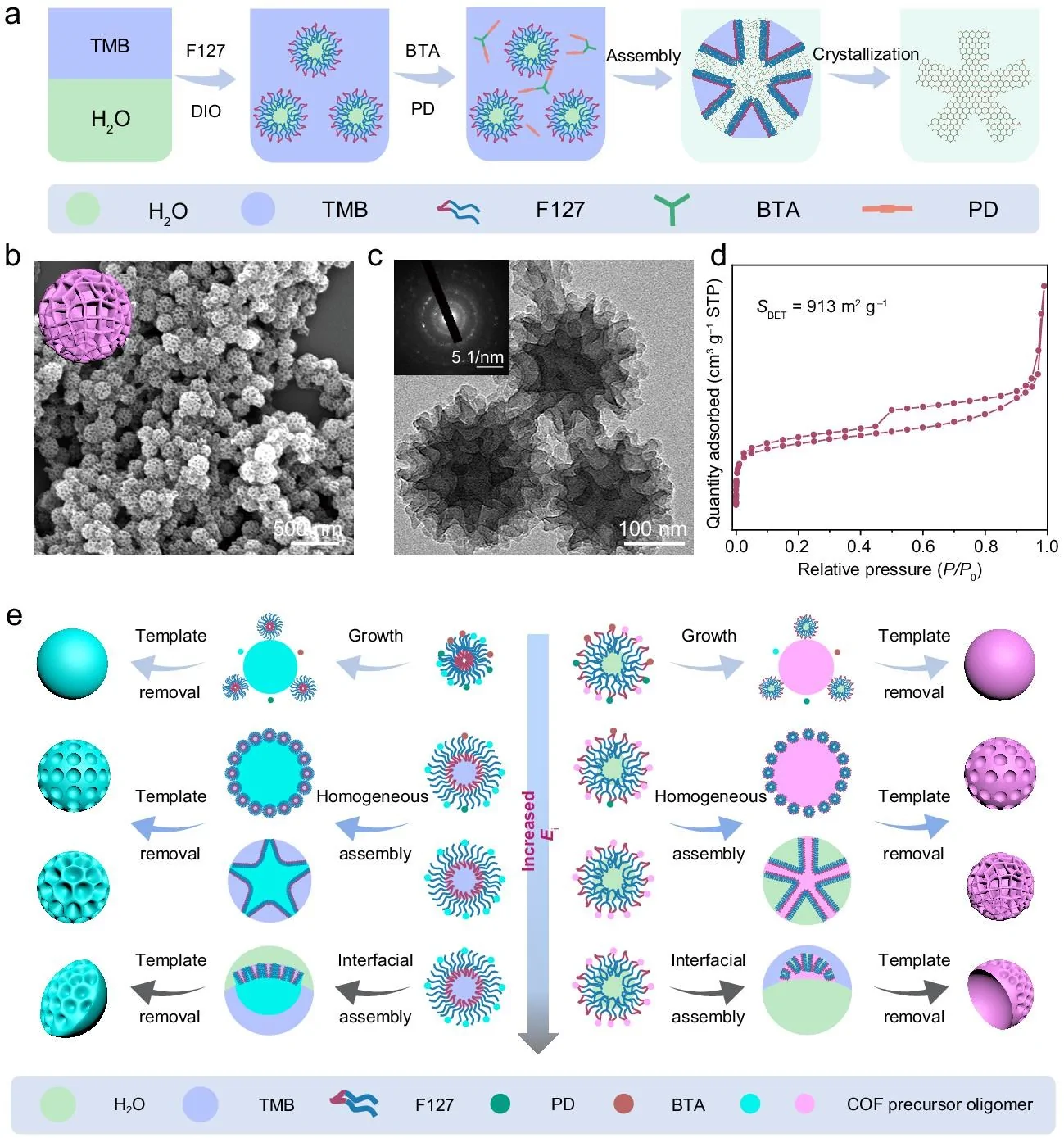
(a) Schematic illustration of the synthesis of dendritic MesoCOFs by reverse emulsion assembly. (b) Scanning electron microscopy image. (c) TEM image. (d) N_2_ sorption isotherm of the dendritic MesoCOFs. (e) Schematic illustration of the formation pathways of MesoCOFs with various architectures. Reproduced from ref. [4] with permission.

Zhao and co-workers [4] now report a pioneering interfacial-energy-driven emulsion-assembly strategy for the facile synthesis of MesoCOFs with diverse symmetries, mesophases and pore sizes. Their strategy employs a water-mesitylene-dioxane-F127 system and a two-step approach: mild emulsion assembly to generate an amorphous mesostructured precursor, followed by solvothermal crystallization that preserves the pre-established pore architecture (Fig. 1a). This elegant decoupling effectively circumvents the trade-off between framework crystallinity and mesostructural order. The resulting MesoCOFs exhibit extraordinary structural diversity, ranging from nanospheres to asymmetric bowls, featuring spherical or dendritic mesophases with pore sizes continuously tunable from 13.2 to 48.5 nm. Scanning electron microscopy (SEM, Fig. 1b), transmission electron microscopy (TEM, Fig. 1c) and N_2_ sorption analysis (Fig. 1d) collectively confirm that the dendritic MesoCOF nanoparticles exhibit uniform spherical morphology (120 nm), radially oriented dendritic mesochannels and hierarchical micro-mesoporous architectures.

The authors demonstrate that interfacial energy scales systematically with the volume fraction of the dispersed phase. An increase in interfacial energy not only swells micelles but also shifts the assembly pathway from a homogeneous mode, in which micelles are uniformly distributed within droplets, to an interfacial mode, in which they preferentially assemble at the oil–water interface. This energy-dependent mechanism establishes a predictive continuum: moderate interfacial energy yields spherical pores; higher energy produces dendritic structures through micelle deformation and fusion; and very high interfacial energy expels micelles to the interface, yielding asymmetric bowls and cones (Fig. 1e). Validation across multiple emulsion compositions establishes interfacial energy as a practical design parameter for MesoCOF synthesis. Leveraging the structural advantages of large, open mesochannels and abundant C=N sites, the dendritic MesoCOF nanospheres function as ionic dividers for Zn anodes, enabling stable cycling for >1340 h at 1.0 mA cm^−2^. Such performance not only underscores the practical utility of the MesoCOF platform but also validates its design principle at the device level.

Looking ahead, fully realizing the potential of MesoCOFs may require the development of tailored composites and single-crystalline structures to specific requirements. Successful interface-confined and emulsion-based assembly strategies in other material systems, from mesoporous polydopamine nanosheets coated on metal-organic framework’s crystal facets [5] to hollow mesoporous metal-organic framework single crystals [6] fabricated by kinetics-mediated micelle assembly, provide valuable guidelines for future COF engineering.

In summary, this work presents a broadly applicable synthetic route to MesoCOFs and lays the foundation for rational optimization and broader energy-related applications. Building on this advance, a next frontier in surfactant- and emulsion-directed MesoCOF synthesis is the creation of single-crystalline MesoCOF architectures. Such materials integrate crystallized COF with mesoscale pore engineering, enabling more precise structure–property correlations and improved transport-controlled functions. Although the present dataset remains modest, correlations between synthesis conditions and architecture reported here provide a foundation for future data collection and artificial-intelligence-assisted prediction of porous COF architectures. More broadly, this work establishes a benchmark for the rational synthesis of hierarchically structured crystalline porous materials, opening new frontiers for COF-based energy storage and related applications.
